# Esophageal Endoscopic Submucosal Dissection Assisted by an Overtube with a Traction Forceps: An Animal Study

**DOI:** 10.1155/2016/3186168

**Published:** 2016-07-27

**Authors:** Ken Ohata, Kuangi Fu, Eiji Sakai, Kouichi Nonaka, Tomoaki Tashima, Yohei Minato, Akiko Ohno, Takafumi Ito, Yosuke Tsuji, Hideyuki Chiba, Makoto Yamawaki, Hideyuki Hemmi, Teruo Nakaya, Junichi Fukushima, Nobuyuki Matsuhashi

**Affiliations:** ^1^Department of Gastroenterology, NTT Medical Center Tokyo, Tokyo 141-8625, Japan; ^2^Department of Endoscopy, Kanma Memorial Hospital, Nasushiobara 325-0046, Japan; ^3^Department of Gastroenterology, Kyorin University School of Medicine, Tokyo 181-8611, Japan; ^4^Department of Gastroenterology, Keiyu Hospital, Yokohama 220-0012, Japan; ^5^Department of Gastroenterology, University of Tokyo, Tokyo 113-8655, Japan; ^6^Department of Gastroenterology, Omori Red Cross Hospital, Tokyo 143-8527, Japan; ^7^Division of Gastroenterology, Department of Internal Medicine, Yokkaichi Municipal Hospital, Yokkaichi 510-0822, Japan; ^8^Department of Gastroenterology, Moriguchi Keijinkai Hospital, Osaka 570-0021, Japan; ^9^Department of Gastroenterology, Yamaga Chuo Hospital, Yamaga 861-0501, Japan; ^10^Department of Diagnostic Pathology, NTT Medical Center Tokyo, Tokyo 141-8625, Japan

## Abstract

Esophageal endoscopic submucosal dissection (ESD) is technically difficult. To make it safer, we developed a novel method using overtube with a traction forceps (OTF) for countertraction during submucosal dissection. We conducted an* ex vivo* animal study and compared the clinical outcomes between OTF-ESD and conventional method (C-ESD). A total of 32 esophageal ESD procedures were performed by four beginner and expert endoscopists. After circumferential mucosal incision for the target lesion, structured as the isolated pig esophagus 3 cm long, either C-ESD or OTF-ESD was randomly selected for submucosal dissection. All the ESD procedures were completed as en bloc resections, while perforation only occurred in a beginner's C-ESD procedure. The dissection time for OTF-ESD was significantly shorter than that for C-ESD for both the beginner and expert endoscopists (22.8 ± 8.3 min versus 7.8 ± 4.5 min, *P* < 0.001, and 11.3 ± 4.4 min versus 5.9 ± 2.5 min, *P* = 0.01, resp.). The frequency and volume of the submucosal injections were significantly smaller for OTF-ESD than for C-ESD (1.3 ± 0.6 times versus 2.9 ± 1.5 times, *P* < 0.001, and 5.3 ± 2.8 mL versus 15.6 ± 7.3 mL, *P* < 0.001, resp.). Histologically, muscular injury was more common among the C-ESD procedures (80% versus 13%, *P* = 0.009). Our results indicated that the OTF-ESD technique is useful for the safe and easy completion of esophageal ESD.

## 1. Introduction

The incidence of esophageal cancer has increased rapidly in both Western and Asian countries [1], although the predominant pathologic types are quite different [2, 3]. Recent technical innovations in endoscopy (e.g., narrow-band imaging) have enabled improvements in the detectability of superficial esophageal neoplasms [4, 5]. Because of the higher complication rates and lower quality of life in patients undergoing an esophagectomy [6], endoscopic resection is now often used for the treatment of such lesions. To reduce the morbidity and mortality rates associated with advanced esophageal cancer, the appropriate management of early cancer is necessary.

Recently, endoscopic submucosal dissection (ESD) has been applied as an alternative to EMR for the removal of superficial esophageal neoplasms, as it provides a much higher en bloc resection rate and reduced rates of local residual tumor and/or recurrence [7, 8]. Although gastric ESD has now spread worldwide, esophageal ESD remains challenging because of the thin and narrow luminal wall in the esophagus. Moreover, the relatively lower incidence of superficial esophageal neoplasms has made it difficult for surgeons, especially Western endoscopists, to become proficient at performing esophageal ESD given the lack of training opportunities and supervision by experts [9]. Submucosal dissection after the completion of the circumferential mucosal incision is considered to be the most challenging step in ESD, as it is often difficult to obtain a good view of the dissection plane in the narrow esophageal lumen because of respiratory and cardiac pulsations. Underestimating the depth of the submucosal layer and inaccurate identification of the cutting line may cause perforation and/or residual tumor. Therefore, the development of safer and more reliable devices as well as modifications of presently used techniques is required.

To facilitate submucosal dissection, adequate tissue traction and better visualization of the lesion are important. Although such conditions can sometimes be achieved with a postural change, it is difficult to maintain a good countertraction constantly. Previously, we reported a case of esophageal ESD in which a safer and faster submucosal dissection was achieved using a novel overtube with traction forceps (OTF-ESD) [10]. Therefore, we conducted an animal model study comparing the safety and clinical outcomes of OTF-ESD and the conventional method (C-ESD). The objective of the present study was to evaluate the feasibility and potential effectiveness of OTF-ESD for possible application in humans.

## 2. Materials and Methods

Using an animal model, we conducted a prospective, randomized controlled trial comparing two different esophageal ESD techniques: the conventional method (C-ESD) and a newly developed method using a novel overtube with traction forceps (OTF-ESD) for submucosal dissection. The present study was planned according to the guidelines of our institutional review board and the Animal Use Committee.

### 2.1. Animal Models

**Figure 1:**
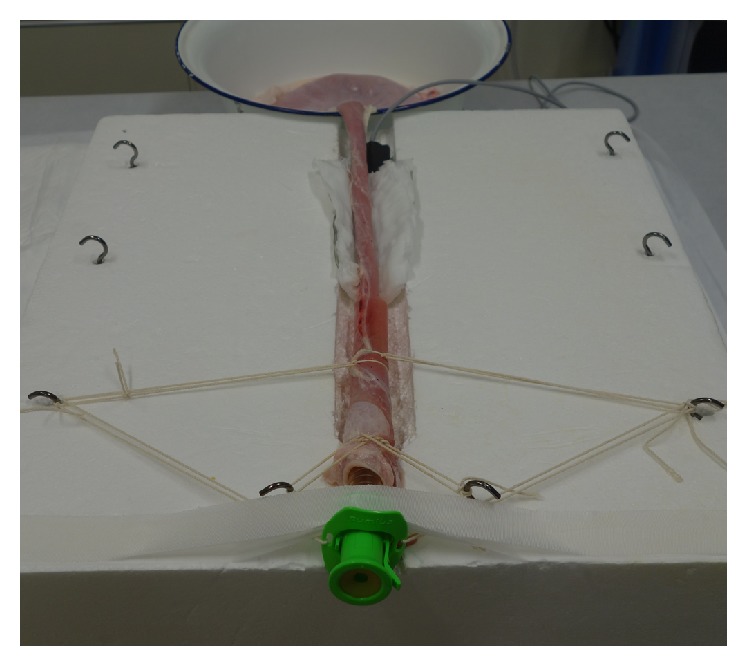
*Ex vivo* porcine esophageal model for endoscopic submucosal dissection.

Isolated pig organs (esophagus, stomach, and duodenum), which were commercially available at a Tokyo meat market, were irrigated with water and Pronase MS (400 U/mL; Kaken Pharma, Tokyo, Japan) before ESD. First, we dug a trench (20 mm in width and 25 cm in length) in a Styrofoam box in which we then placed the pig esophagus; the box was then fixed to a plastic stand (Johnson & Johnson, Tokyo, Japan). We inserted an overtube (Flexible Overtube MD-48518; Sumitomo Bakelite, Tokyo, Japan) into the oral edge of the pig esophagus and tied it firmly with a string. The gastric outlet was also closed tightly with a string to enable air insufflation during ESD. A counter electrode board was placed on top of the bottom wall of the trench. The porcine esophagus was dipped in saline to enable the conduction of electrical current and was then placed inside the trench. Finally, the oral side of the overtube was fixed with gum tape (Figure 1).

### 2.2. ESD Operators

A total of eight endoscopists (T. N., M. Y., H. H., A. O., T. I., H. C., Y. T., and K. O.) were enrolled in this study. The endoscopists were classified into two groups: four beginners who had limited experience performing esophageal ESD procedures and four experts with experience performing more than 30 esophageal ESD procedures. The profile of each operator is shown in Table 1. Each endoscopist resected four lesions: two using C-ESD and two using OTF-ESD.

### 2.3. Devices and Settings

In this study, an upper GI endoscope (GIF-Q260J; Olympus Optical Co., Tokyo, Japan) with a transparent attachment cap (D201-10704, 4 mm; Olympus Optical Co.) was used for all the procedures. A dual knife (KD-650L; Olympus Optical Co.) was used for the circumferential mucosal resection, and the submucosal dissection was mainly performed using an IT knife nano (KD-612L; Olympus Optical Co.). A VIO generator (VIO300D; Erbe Elektromedizin, Tubingen, Germany) was used for all the ESD procedures as a high-frequency generator unit. The settings were as follows: Endocut I Effect 3 (duration 2, interval 2), 60 W for all ESD procedures, and Swift Coagulation, 45 W for submucosal dissection. Submucosal injection was performed using Glyceol mixed with indigo carmine using a 23-gauge, 4 mm long needle (Olympus, Optical Co).

### 2.4. Design and Concept of OTF Method

**Figure 2:**
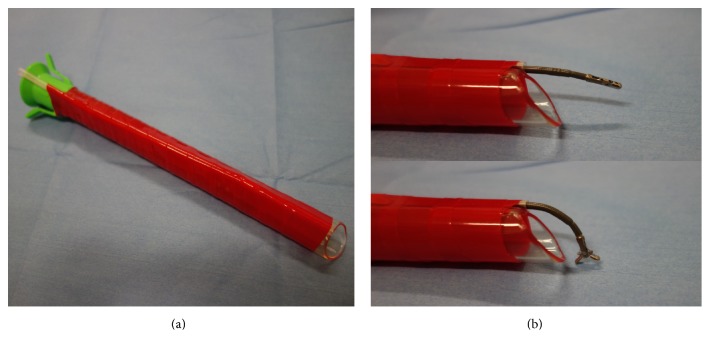
Design of overtube with traction forceps. (a) To create an accessory work channel, a long straw tube was mounted with red vinyl tape on an overtube. (b) The grasping forceps were modified so that they were curved when opened, allowing the edges of the target lesion to be grasped and dissected.

To create an accessory work channel, we mounted a long straw tube with red vinyl tape on an overtube (Figure 2(a)). Next, we modified a pair of grasping forceps (FB-230K; Olympus, Optical Co.) in a way such that they become straight when closed and curved when opened, enabling the edge of the target lesion to be grasped during the submucosal dissection (Figure 2(b)). Importantly, the modified overtube can be rotated around the endoscope within the esophagus, thereby enabling the target lesion to be regrasped from 360 degrees, if needed. Using the OTF method, adequate tissue traction and better visualization were expected to be maintained constantly.

### 2.5. ESD Procedure

The target lesion was created by endoscopic marking using an electrosurgical knife; each target lesion covered half the circumference of the esophageal lumen and measured 3 cm in length. After first ESD procedure, the next target lesion was created at 5 cm proximal to the previous resected margin. A total of two target lesions were removed from each porcine* ex vivo* animal model. After the circumferential mucosal resection, each of the two lesions was randomized into either a C-ESD group or an OTF-ESD group for submucosal dissection.

**Figure 3:**
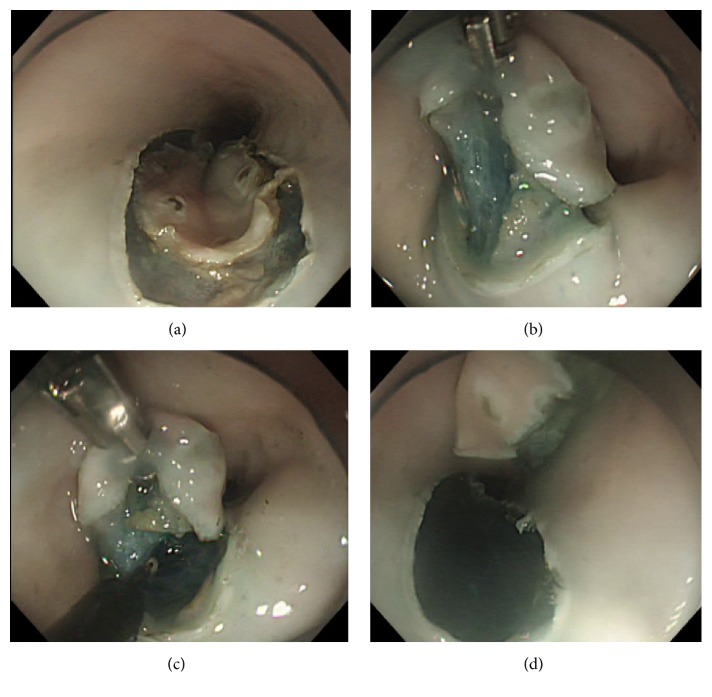
Submucosal dissection using an overtube with traction forceps (ESD). (a) The circumferential mucosal incision for the target lesion was completed. (b) The target lesion was grasped with the forceps and pulled to the luminal side, enabling the dissection plane to be clearly visualized. (c) Under adequate tissue traction, the submucosal dissection was performed using an IT knife. (d) The ESD procedure was completed as en bloc resection.

The C-ESD procedure was conducted as previously described [8]. OTF-ESD was performed according to the following steps. First, a solution was injected into the submucosal layer, similar to the step used in the C-ESD procedure (Figure 3(a)). Then, an assistant operator inserted the modified grasping forceps into the accessory channel on the overtube and grasped the proximal edge of the target lesion; the assistant then straightened the forceps and lifted the submucosal layer. Adjusting the rotation angle of the overtube, the mucosal flap was retracted opposite to the surgical bed, enabling the submucosal layer to be clearly visualized without additional injections (Figure 3(b)). While the assistant continued to lift the mucosa with the forceps, the submucosal dissection was performed (Figure 3(c)). During the procedure, the assistant regulated and adjusted the direction of the lifted layer as appropriate, and the target lesion was finally removed (Figure 3(d)).

### 2.6. Outcomes and Data Analysis

The main outcome of the present study was the dissection time, which was defined as the time between the first submucosal injection of solution until the complete removal of the target lesion. In addition, the completion and en bloc resection rates, specimen size, perforation rate, and volume and frequency of injection needed for submucosal dissection were also evaluated as secondary outcomes. These measurements were compared between the OTF-ESD and C-ESD groups. The muscular layer of the dissected areas was fixed in 10% formalin and embedded in paraffin. After staining with hematoxylin and eosin, the depth of the muscular layer injury was histologically evaluated by a pathologist who was blinded to the ESD technique. When injury to the outer muscular layer (deeper half of the muscularis propria) was observed, the ESD procedure was evaluated as being positive for muscular layer injury.

### 2.7. Statistical Analysis

Continuous data are shown as the mean ± SD. Categorical variables were compared using the Fisher exact test. Continuous variables were compared using the Student *t*-test. Unless otherwise specified, *P* values of <0.05 were considered to denote statistical significance. All the statistical analyses were performed using PASW Statistics 18 for Windows (SPSS Japan, Tokyo, Japan).

## 3. Results

A total of 32 ESD procedures (16 C-ESD and 16 OTF-ESD) were performed using 16* ex vivo* porcine models. The outcomes were summarized in Table 2.

### 3.1. Performance of ESD Procedure

All the ESD procedures were completed as en bloc resections. Although perforation occurred in one beginner's C-ESD procedure, no perforations occurred during the OTF-ESD procedures. No significant difference in the measured area of the resected lesions was seen between the C-ESD and OTF-ESD groups (13.3 ± 1.2 cm^2^ versus 13.6 ± 1.5 cm^2^, *P* = 0.51).

### 3.2. Dissection Time Needed for Submucosal Dissection

**Figure 4:**
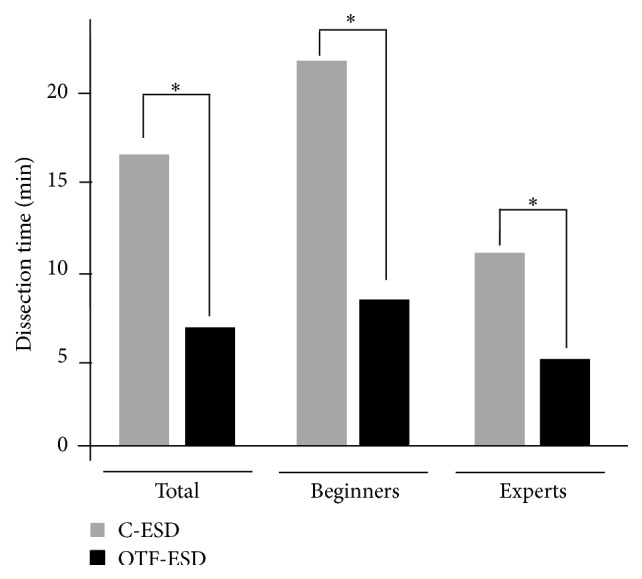
Comparison of dissection times between C-ESD and OTF-ESD groups. The dissection time required for OTF-ESD was significantly shorter than that required for C-ESD (*P* < 0.001). The same tendency was observed not only in the expert group but also in the beginner group (*P* = 0.01 and *P* < 0.001, resp.). ^*∗*^ *P* value with statistical significance.

The dissection time for OTF-ESD was significantly shorter than that for C-ESD (17.0 ± 8.8 min versus 6.8 ± 3.8 min, *P* < 0.001). Interestingly, the same tendency was observed in both the expert and the beginner group (11.3 ± 4.4 min versus 5.9 ± 2.5 min, *P* = 0.01, and 22.8 ± 8.3 min versus 7.8 ± 4.5 min, *P* < 0.001, for the expert and beginner groups, resp.) (Figure 4).

### 3.3. Volume and Frequency of Submucosal Injections

The frequency of submucosal injection was significantly lower for the OTF-ESD group than for the C-ESD group (1.3 ± 0.6 times versus 2.9 ± 1.5 times, *P* < 0.001), and the volume of the submucosal injections was also significantly smaller for the OTF-ESD group (5.3 ± 2.8 mL versus 15.6 ± 7.3 mL, *P* < 0.001). Significant differences were also seen in both the beginner and expert groups (Table 2).

### 3.4. Histological Evaluation

A total of 12 thermal muscular layer injuries were confirmed histologically: 10 occurred during C-ESD, and 2 occurred during OTF-ESD (Table 3). Thermal muscular layer injury to the ulcer floor was significantly less common in the OTF-ESD group (80% versus 13%, *P* = 0.009).

## 4. Discussion

Using an* ex vivo* animal model, we demonstrated the effectiveness of OTF-ESD. Our results indicated that adequate tissue traction using a newly developed overtube enabled both beginner and expert endoscopists to complete esophageal ESD safely and easily.

ESD has been an emerging technique for the treatment of superficial esophageal neoplasms, while the EMR technique is mainly used in Western countries. Although EMR can be effective for removing small localized superficial esophageal neoplasms, the en bloc resection of lesions larger than 20 mm can be difficult; such difficulties can lead to incorrect pathological examinations and a relatively high frequency of local recurrence [11]. The main advantage of ESD is the higher rate of complete resection, regardless of tumor size. Until now, the efficacy and safety of esophageal ESD have been evaluated mainly in Asian countries [12]. Recently, Probst et al. reported that ESD is effective for the treatment of early esophageal adenocarcinoma, as well as squamous cell carcinoma [13]. Meanwhile, Neuhaus et al. reported a relatively low complete en bloc resection rate for early esophageal adenocarcinoma (38.5%) [14], indicating that surgeons in Western countries are still acquiring proficiency in ESD. To overcome the technical difficulties associated with esophageal ESD and to establish this technique as a standard treatment worldwide, technical innovations are required.

Several reports have attempted to make the ESD procedure easier and safer using various traction devices [15–23]. Tsao et al. reported the usefulness of a modified fish-line traction for the ESD of large superficial esophageal cancer [15]. Chen et al. conducted an animal study and demonstrated the efficacy and safety of percutaneous transgastric traction-assisted esophageal ESD, which was designed to offer esophageal mucosal traction directed to the stomach [24]. Our method is different from those used by other devices from the following viewpoints. First, the traction is totally independent of the endoscope's movement. Therefore, our method is especially useful for the tumors located at the left side wall of the esophagus, where the fluid and blood tend to be pooled. Second, the target tissue can be repeatedly grasped easily and effectively, if needed. Third, various traction directions, such as a left/right direction and an oral/anal direction, can be obtained by rotating the overtube and pulling/pushing the grasping forceps, respectively. The length of the flexible overtube is approximately 20 cm. Although operators should pull the overtube slightly during ESD procedure, the lesions located at cervical/upper esophagus could be also treated using OTF method. These advantages contribute to maintaining an adequate traction and stable visibility of the submucosal layer to facilitate a safer and faster submucosal dissection in various situations.

Recently, Hirota et al. conducted an* in vivo* animal study and evaluated the efficacy of a full-traction esophageal ESD technique using a similar overtube [25]. They reported that the procedure time did not differ between the conventional technique and full-traction ESD, while the frequency of submucosal injection was significantly reduced by the full-traction technique. In contrast to their results, we found that the dissection time was significantly reduced by the OTF method. In addition, we also demonstrated that muscular layer injury to the ulcer floor was significantly less common in the OTF-ESD group. These discrepancies can probably be explained by differences in lesion size, animal model, cutting device, and setting. At our institution, submucosal dissection was performed using the “cut mode” once an appropriate dissection line had been confirmed; this factor may be associated with our improved dissection time. Importantly, our results indicated that OTF-ESD can enable a faster and safer ESD for beginners as well as experts. The performance of ESD is thought to be highly dependent on the expertise of the operator. Tsou et al. reported that at least 30 esophageal ESD procedures are needed for a novice endoscopist to gain proficiency in this technique [26]. Technical difficulties such as uncontrollable hemorrhage frequently occur during submucosal dissection and can result in unwanted perforation. As the OTF method helps to maintain a good view of the dissection plane and may reduce such situations, it can offer opportunities for novice ESD operators to achieve higher self-completion rates.

The present study had several limitations. First, this study was a single-centered, preliminary study with a relatively small sample size. Second, an operator should consider the risk of overtube-associated complications, such as perforation, whereas the prevalence is reportedly quite low [27]. Third, the study used an* ex vivo* animal model in which hemostasis was not required during the procedure, in addition to the lack of esophageal movement induced by peristalsis, breathing, and heartbeat. However, as the OTF method provides better fixation and visualization of the submucosal layer during submucosal dissection, we strongly believe that it might also facilitate dissection in living animals and/or humans. Finally, no data is available on esophageal ESD performed in humans by endoscopists who have received training using this model. Further prospective human studies are needed to demonstrate the efficacy and safety of the OTF method for esophageal ESD.

## 5. Conclusion

We revealed the potential advantages of the OTF method for performing safe and easy esophageal ESD. The OTF method is useful not only for experts but also for beginners, thus potentially contributing to the achievement of preferable outcomes for esophageal ESD worldwide and offering an opportunity to overcome the present learning curve.

## Figures and Tables

**Table 1:** Profile of each ESD operator enroll1ed in this study.

	Beginners				Experts			
	T. N.	M. Y.	H. H.	A. O.	T. I.	H. C.	Y. T.	K. O.
Endoscopic experience, years	9	8	10	5	5	6	6	12
Gastric ESDs performed, number	5	30	30	50	150	150	200	1500
Esophageal ESDs performed, number	0	2	0	1	50	50	30	150

Beginners were defined as operators with limited experience performing esophageal ESDs. Experts were defined as operators who had performed more than 30 esophageal ESDs and more than 150 gastric ESDs.

**Table 2:** Outcomes of esophageal ESD according to C-ESD and OTF-ESD.

	C-ESD			OTF-ESD			^*∗*^ *P* values		
	Total	Beginners	Experts	Total	Beginners	Experts	Total	Beginners	Experts
Number of lesions	16	8	8	16	8	8			
Total dissection time, min	17.0 ± 8.8	22.8 ± 8.3	11.3 ± 4.4	6.8 ± 3.8	7.8 ± 4.5	5.9 ± 2.5	<0.001	<0.001	0.01
Lesion size, cm^2^	13.3 ± 1.2	13.1 ± 0.9	13.4 ± 1.5	13.6 ± 1.5	13.2 ± 1.4	14.0 ± 1.6	0.51	0.88	0.49
Self-completion, *n* (%)	16 (100)	8 (100)	8 (100)	16 (100)	8 (100)	8 (100)	n.a.	n.a.	n.a.
En bloc resection, *n* (%)	16 (100)	8 (100)	8 (100)	16 (100)	8 (100)	8 (100)	n.a.	n.a.	n.a.
Perforation, *n* (%)	1 (6)	1 (13)	0 (0)	0 (0)	0 (0)	0 (0)	>0.99	>0.99	n.a.
Frequency of submucosal injection, *n*	2.9 ± 1.5	3.5 ± 1.4	2.3 ± 1.2	1.3 ± 0.6	1.6 ± 0.5	1.0 ± 0.0	<0.001	0.005	0.02
Volume of submucosal injection, mL	15.6 ± 7.3	18.5 ± 7.3	13.8 ± 6.0	5.3 ± 2.8	6.3 ± 3.1	4.4 ± 2.2	<0.001	0.001	0.004

C-ESD, conventional endoscopic submucosal dissection; OTF-ESD, overtube with forceps endoscopic submucosal dissection.

Continuous data are shown as the mean ± SD.

^*∗*^ *P* values were compared between the C-ESD and OTF-ESD groups.

**Table 3:** Histological evaluation of thermal injury to the muscular layer.

	C-ESD	OTF-ESD
Muscular layer injury (+)	10 (63%)	2 (13%)
Muscular layer injury (−)	6 (38%)	14 (88%)

Abbreviations: C-ESD, conventional endoscopic submucosal dissection; OTF-ESD, overtube with forceps endoscopic submucosal dissection.

NOTE: When injury of the outer muscular layer was observed, the ESD procedure was evaluated as positive for muscular layer injury.

*P* = 0.009, calculated using the Fisher exact test.
